# Radiotherapy-Resistant Breast Cancer Cells Enhance Tumor Progression by Enhancing Premetastatic Niche Formation through the HIF-1α-LOX Axis

**DOI:** 10.3390/ijms21218027

**Published:** 2020-10-28

**Authors:** Young Shin Ko, Trojan Rugira, Hana Jin, Young Nak Joo, Hye Jung Kim

**Affiliations:** 1 Department of Pharmacology, Institute of Health Sciences, College of Medicine, Gyeongsang National University, Jinju 52727, Korea; shini33@naver.com (Y.S.K.); rugirawacu@gmail.com (T.R.); hanajin.kr@daum.net (H.J.); hahaha-_-0001@hanmail.net (Y.N.J.); 2 Department of Convergence Medical Science, Gyeongsang National University, Jinju 52727, Korea

**Keywords:** cancer stem cell, chemotherapy resistance, radio-resistant breast cancer cell, premetastatic niche formation

## Abstract

Cancer stem cells (CSCs) exist in solid tumors and contribute to therapeutic resistance and disease recurrence. Previously, we reported that radiotherapy-resistant (RT-R)-MDA-MB-231 cells from highly metastatic MDA-MB-231 cells produced more CSCs than any other RT-R-breast cancer cells and showed therapeutic resistance and enhanced invasiveness. Hypoxia inducible factor-1α (HIF-1α) induced in the tumor microenvironment leads to the release of lysyl oxidase (LOX), which mediates collagen crosslinking at distant sites to facilitate environmental changes that allow cancer cells to easily metastasize. Therefore, in this study, we investigated whether RT-R-MDA-MB-231 cells induce greater HIF-1α expression, LOX secretion, and premetastatic niche formation than MDA-MB-231 cells do. RT-R-MDA-MB-231 cells increased HIF-1α expression and LOX secretion compared with MDA-MB-231 cells. Mice harboring RT-R-MDA-MB-231 cell xenografts showed enhanced tumor growth and higher expression of the CSC markers, CD44, Notch-4, and Oct3/4. In addition, mice injected with RT-R-MDA-MB-231 cells exhibited a higher level of HIF-1α in tumor tissue, increased secretion of LOX in plasma, higher induced levels of crosslinked collagen, and a higher population of CD11b^+^ BMDC recruitment around lung tissue, compared with those injected with MDA-MB-231 cells. These results suggest that RT-R-MDA-MB-231 cells contribute to tumor progression by enhancing premetastatic niche formation through the HIF-1α-LOX axis.

## 1. Introduction

It is known that most deaths in cancer patients, including breast cancer, are due to invasion, metastasis of the primary tumor to distant organs, and recurrence of the tumor [1,2]. Generally, breast cancer patients are treated with radiotherapy, surgery, chemotherapy, hormone therapy, or target therapy [3]. Even though radiotherapy is an important treatment for breast cancer patients, some develop tumor recurrence after radiotherapy. Recently, cancer stem cells (CSCs) have been found to exist in tumors and lead to metastasis to distant organs, therapy resistance, and disease recurrence. Some or all CSCs are drivers of tumor progression and are supported by specific microenvironmental niches. The tumor microenvironment contributes to the maintenance and regeneration of CSCs [4,5] and renders CSCs more resistant to radiotherapy and chemotherapy, ultimately resulting in tumor regrowth and distant metastasis [6,7,8,9,10] by mediating interactions between tumor cells, their secreted factors, and the endothelium [11,12]. Recently, it has been proposed that the number of CSCs in tumor populations is not fixed, but the transition between CSCs and non CSCs occurs in response to the tumor environment and treatment, indicating the importance of CSC phenotype on tumor behavior [13].

In order to verify the prognostic value and usefulness of CSCs in the monitoring of therapeutic efficacy, many attempts have been made to better characterize and identify CSCs. First, Al-Hajj et al. [14] found that CD44^+^/CD24^−^ breast cancer cells represent distinct characteristics of CSCs, and then several surface markers such as aldehyde dehydrogenase (ALDH), EpCAM, and LGR5 have been reported [15,16]. In addition, it was suggested that pathways involved in stemness and self-renewal such as Wnt [17], PI3K/Akt/FOXO [18], TGF-β [19], and Notch [20] are dysregulated in CSCs. Furthermore, Oct4, also known as Oct-3, which belongs to the POU (Pit-Oct-Unc) transcription factor family [21], has been suggested as a CSC marker based on the reports that high Oct4 activity can be used to identify a cancer stem-like cell from breast carcinoma [22]. Oct4 has also been shown to increase tumor growth, invasion, and chemoresistance, and dedifferentiate differentiated head and neck squamous carcinoma cells to CSC-like cells [23].

In our previous study [24], we established radiotherapy-resistant (RT-R) breast cancer cells by repeated irradiation (2 Gy each, 25 times, total 50 Gy; this is used clinically to treat breast cancer patients) and reported that RT-R-MDA-MB-231 cells derived from highly metastatic MDA-MB-231 cells showed the most radio- and chemo-resistance of the three tested cell lines (RT-R-MDA-MB-231, RT-R-MCF-7, RT-R-T47D). When we compared the response between MDA-MB-231 cells and RT-R-MDA-MB-231 cells to irradiation, irradiation reduced cell viability of MDA-MB-231 cells by about 33% compared to non-irradiated MDA-MB-231; however, RT-R-breast cancer cells exhibited about 50% greater resistance than parental MDA-MB-231. From these results, we confirmed the successful establishment of RT-R-MDA-MB-231 cells by repeated irradiation. Furthermore, we found that RT-R-MDA-MB 231 cells increased protein levels of the CSC markers CD44, Notch-4, Oct3/4, and ALDH1 more than MDA-MB-231 cells [24].

The acquired radiotherapy-resistance can be due to the presence of hypoxia in the tumor environment [25]. It has been reported that CSCs depend largely on hypoxia inducible factors (HIFs) for survival, self-renewal, and tumor propagation [26]. Our previous study also reported that the expression of HIF-1α, leading to the secretion of lysyl oxidase (LOX), is increased in the highly metastatic breast cancer cells MDA-MB-231 [27]. In addition, clinical studies have shown that both HIF-1α and LOX are overexpressed in breast cancer patients, and this overexpression increases with disease progression and is associated with a high mortality rate [28,29]. Accordingly, in this study, we hypothesized that in a tumor microenvironment, RT-R-MDA-MB-231 cells, which contain more CSCs than MDA-MB-231 cells, induce HIF-1α expression and LOX secretion, and then finally premetastatic niche formation, more than MDA-MB-231 cells do.

## 2. Results

### 2.1. RT-R-MDA-MB-231 Cells Showed Morphological Changes and Induced Colony Forming Ability and Proliferation

**Figure 1:**
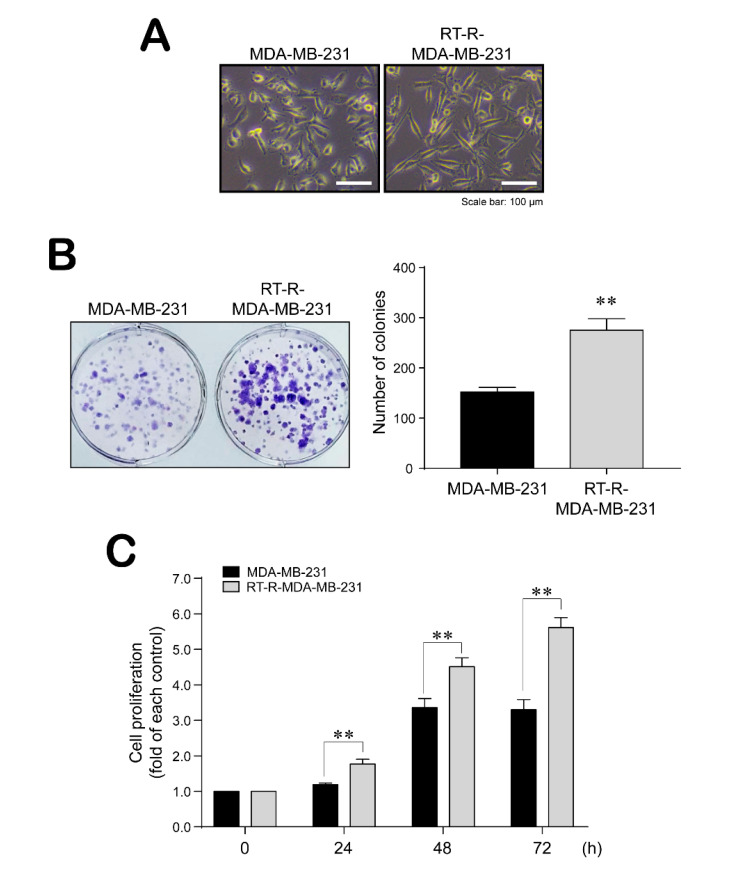
Radiotherapy-resistant (RT-R)-MDA-MB-231 cells showed morphological changes and enhanced colony formation and proliferation. (**A**) The morphologic changes of cells were observed under a light microscope (×200). (**B**) The colony formation assay was performed as described in the Methods. Cells were seeded at 10^3^ cells in 6-well plates, and 10 days later, colonies were stained with 0.1% Giemsa staining solution. Data represent mean values ± SEM of three independent experiments. Significance compared with MDA-MB-231, ** *p* < 0.01. (**C**) Cells were seeded at 10^4^ cells in 24-well plates and incubated for 24, 48, and 72 h. Cell proliferation was determined by 3-(4,5-dimethylthiazol-2-yl)-2,5-diphenyltetrazolium bromide (MTT) assay as described in the Methods. Data represent mean values ± SEM of three independent experiments. ** *p* < 0.01.

We first compared the morphology between MDA-MB-231 cells and RT-R-MDA-MB-231 cells and found that the RT-R-MDA-MB-231 cells changed into a more mesenchymal form with a slightly longer shape compared with their parent MDA-MB-231 cells (Figure 1A). In addition, we examined the ability of MDA-MB-231 and RT-R-MDA-MB-231 cells to proliferate and form colonies. As expected, RT-R-MDA-MB-231 cells showed increased colony forming ability and proliferation compared with MDA-MB-231 cells (Figure 1B,C).

### 2.2. RT-R-MDA-MB-231 Cells Upregulated HIF-1α Expression and LOX Secretion

**Figure 2:**
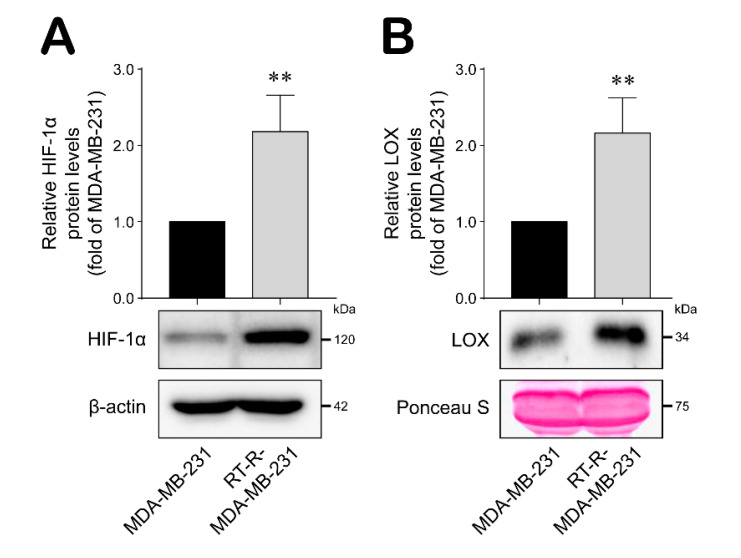
HIF-1α expression and lysyl oxidase (LOX) secretion were increased in RT-R-MDA-MB-231 cells compared with MDA-MB-231 cells. (**A**,**B**) HIF-1α expression level (**A**) and secreted LOX level (**B**) were detected from cell lysates and medium, respectively, by western blot analysis as described in the Methods. Data represent mean values ± standard error (SEM) of three independent experiments. Significance compared with MDA-MB-231, ** *p* < 0.01.

It was recently reported that CSCs are induced and maintained by the tumor microenvironment, especially the hypoxic conditions that are found there [30,31]. Under hypoxic conditions, LOX is secreted to mediate premetastatic niche formation and promote cancer metastasis [27]. Thus, we analyzed HIF-1α expression and LOX secretion in RT-R-MDA-MB-231 cells. As shown in Figure 2, HIF-1α expression and LOX secretion were significantly induced in RT-R-MDA-MB-231 cells compared with MDA-MB-231 cells, supporting the existence of hypoxic conditions and suggesting the increase of radio-resistant CSCs within the RT-R-MDA-MB-231 cell population.

### 2.3. Mice with RT-R-MDA-MB-231 Xenografts Exhibited Increased Tumor Progression and Higher Level of CSC Markers CD44, Notch-4, and Oct3/4, But Not ALDH1

**Figure 3:**
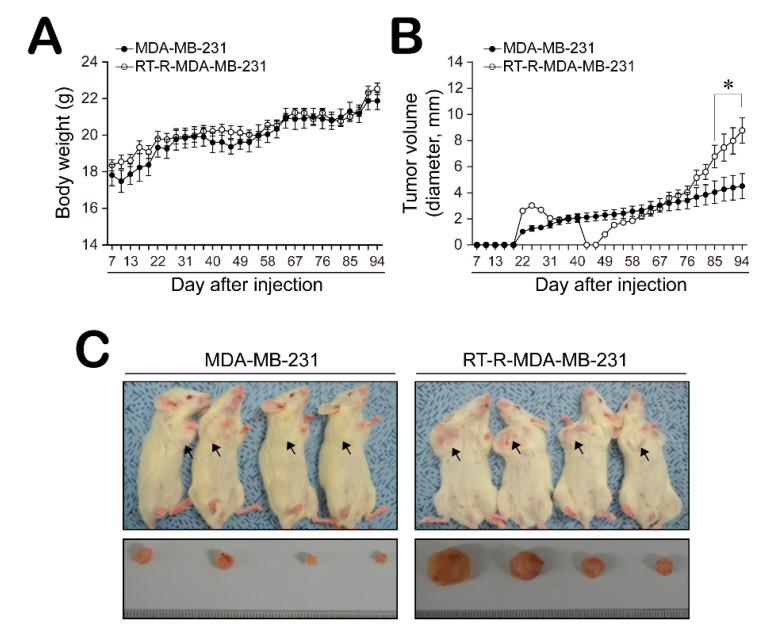
RT-R-MDA-MB-231 cells promoted tumor growth in an in vivo mouse model. Female non obese diabetic-severe combined immunodeficiency (NOD-SCID)mice were divided into two groups and injected subcutaneously into the mammary fat pad with MDA-MB-231 cells and RT-R-MDA-MB-231 cells (5 × 10^6^ cells/100 μL of serum-free medium), respectively. Body weights (**A**) and tumor volumes (**B**) were measured every 3 days during tumor development. MDA-MB-231-injected or RT-R-MDA-MB-231-injected animals were sacrificed at day 90 after injection, and the tumors were extracted (**C**) (* *p* < 0.05, compared between MDA-MB-231- and RT-R-MDA-MB-231-injected groups).

**Figure 4:**
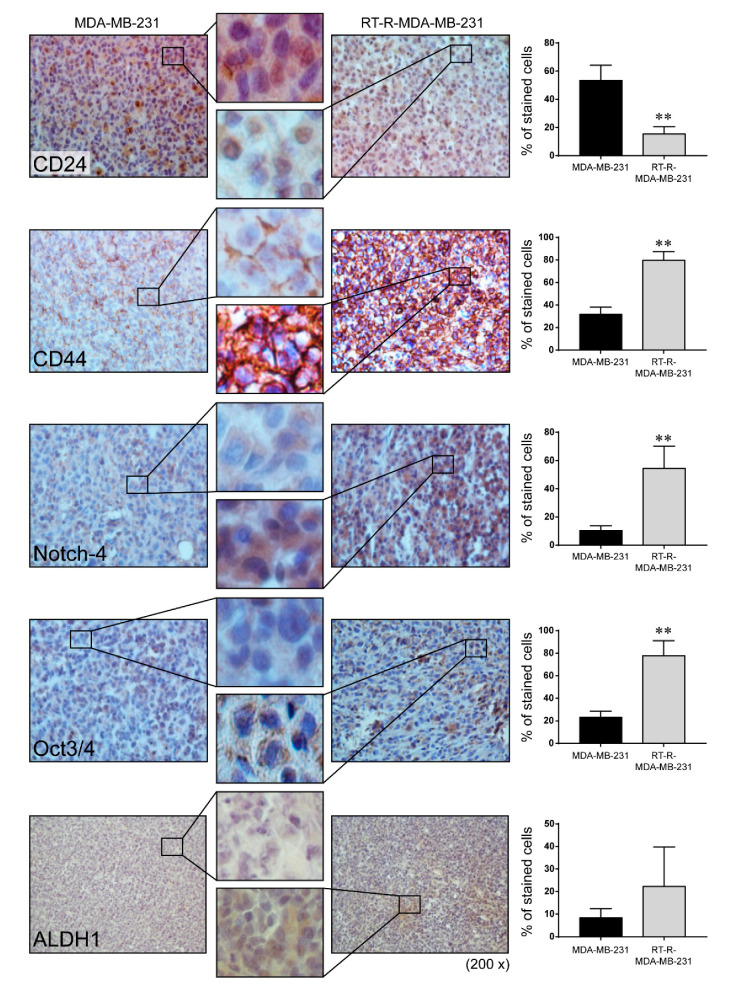
Cancer stem cell markers CD44, Notch-4, and Oct3/4 but not ALDH1 were highly detected in the tumor tissues from RT-R-MDA-MB-231 cell-injected mice. Cancer stem cell markers including CD24, CD44, Notch-4, Oct3/4, and ALDH1 were detected in the tumor sections using the specific antibodies as described in the Methods. The sections were counterstained with hematoxylin and examined under a light microscope. The representative images were presented (magnification; ×200 and ×400). Data represent mean values ± SEM (*n* = 10). ** *p* < 0.01 compared with the MDA-MB-231-injected mice.

Finally, we confirmed whether RT-R-MDA-MB-231 cells increase tumor growth or invasiveness using an in vivo animal model. Female non obese diabetic-severe combined immunodeficiency (NOD-SCID) mice were divided into two groups and either MDA-MB-231 or RT-R-MDA-MB-231 cells were injected subcutaneously into the mammary fat pad. The body weights and tumor volumes were measured every 3 days for 90 days. There was no significant change in body weight (Figure 3A). Interestingly, tumors were detectable by the 16th day after injection for both MDA-MB-231 and RT-R-MDA-MB-231 xenografts; however, the RT-R-MDA-MB-231 xenografts seemed to disappear and then rapidly regrow, whereas the MDA-MB-231 tumors showed steady growth (Figure 3B). Figure 3C showed that the tumor volume at the end of 90 days was significantly increased in the RT-R-MDA-MB-231 group. In addition, immunohistochemistry (IHC) staining revealed that RT-R-MDA-MB-231 xenograft tissue showed a significantly higher expression of CSC markers, such as CD44, Notch-4, and Oct3/4 (Figure 4). In the case of ALDH1, its expression level looked a little higher in the mice injected with RT-R-MDA-MB-231 cells compared to those injected with MDA-MB-231, but this was not significant.

### 2.4. Mice Injected with RT-R-MDA-MB-231 Cells Showed Increased HIF-1α Expression Level, LOX Secretion, and Collagen Crosslinking and BMDC Recruitment to the Lungs Compared with Those Injected with MDA-MB-231 Cells

**Figure 5:**
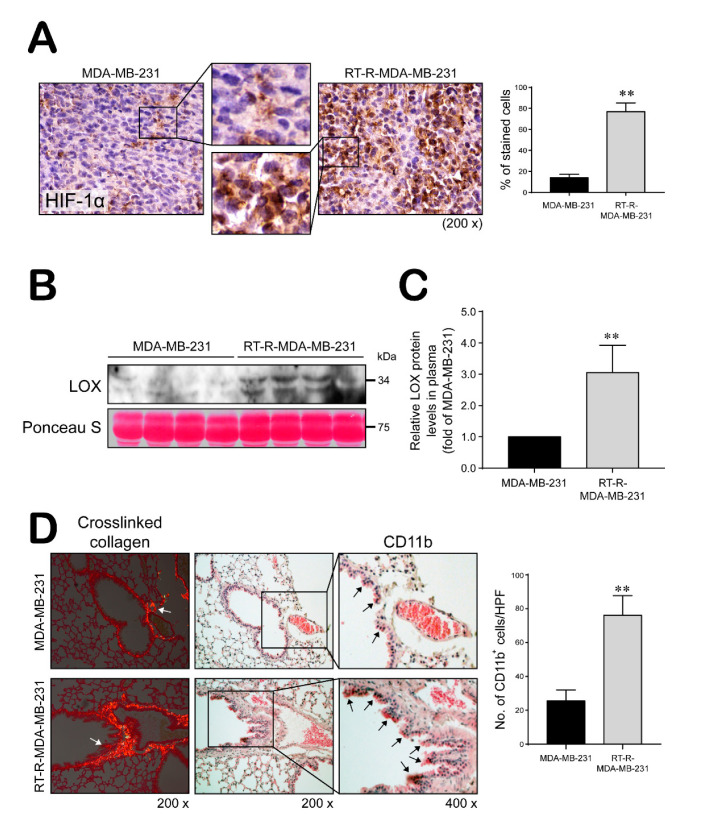
RT-R-MDA-MB-231 cell-injected mice increased HIF-1α levels and LOX secretion, and premetastatic niche formation. (**A**) HIF-1α levels detected in tumor section by immunohistochemistry (magnification; ×200 and ×400). Data represent mean values ± SEM (*n* = 10). ** *p* < 0.01 compared with the MDA-MB-231-injected mice. (**B**,**C**) Secreted LOX levels were detected in the plasma by western blot analysis. Data represent mean values ± SEM (*n* = 8). ** *p* < 0.01 compared with the MDA-MB-231-injected mice. (**D**) Lung tissue sections from MDA-MB-231- and RT-R-MDA-MB-231-injected mice were stained with Picrosirius Red to analyze crosslinked collagen. Recruited CD11b^+^-immunoreactive bone marrow-derived dendritic cells (BMDCs) near the sites of collagen cross-linkage were stained with anti-CD11b antibody. White arrows indicate representative crosslinked collagen fibers. Black arrows indicate recruited CD11b^+^ BMDCs (magnification; ×200 and ×400). Data represent mean values ± SEM (*n* = 10). ** *p* < 0.01 compared with the MDA-MB-231-injected mice.

Mice bearing RT-R-MDA-MB-231 xenografts expressed a significantly higher level of HIF-1α in tumor tissue and significantly increased plasma LOX levels compared to those with MDA-MB-231 cells (Figure 5A–C). Moreover, higher levels of crosslinked collagen were detected in lung sections from RT-R-MDA-MB-231-injected mice compared with those injected with MDA-MB-231 cells (Figure 5D). Additionally, RT-R-MDA-MB-231-injected mice showed greater recruitment of CD11b^+^ bone marrow-derived dendritic cells (BMDCs) around the sites of crosslinked collagen in the lungs compared with MDA-MB-231-injected mice (Figure 5D), suggesting that RT-R-MDA-MB-231 cells stimulate premetastatic niche formation.

## 3. Discussion

Combination therapy, which consists of surgery, chemotherapy, and radiotherapy, is used to treat cancer patients, including breast cancer patients. In addition to surgery and systemic therapy, radiotherapy is regarded as a crucial treatment option in modern cancer therapy as it leads to improvement in overall survival [32,33,34]. However, radiotherapy has limitations that can lead to therapeutic failure. Tumor recurrence after radiotherapy is common, and once the disease recurs, it readily metastasizes to distant organs and causes death. In order to metastasize to distant locations, the receptive microenvironments must be prepared for the development of cancer cells. A premetastatic niche is a fertile microenvironment that forms in a metastatic target organ and facilitates the promotion of metastasis [35]. Prior recruitment of monocytes to crosslink collagen at distant sites facilitates environmental changes that promote cancer cell colonization and metastasis [36,37,38].

Recent studies suggested that CSCs exist in solid tumors and contribute to therapeutic resistance and disease recurrence. In our previous study, we also reported that RT-R-breast cancer cells increased the population of CSCs. In particular, RT-R-MDA-MB-231 derived from highly metastatic breast cancer cells produced more CSCs and also acquired resistance to other cancer therapies, as well as radiotherapy. However, the role of CSCs in premetastatic niche formation is unknown. As we mentioned before, the presence of hypoxia in the tumor environment can generate CSCs and cause acquisition of radiotherapy-resistance [25], and under hypoxic conditions, HIF-1α and LOX are stabilized and secreted, respectively, and mediate premetastatic niche formation to promote cancer metastasis [39]. Therefore, in this study, we chose RT-R-MDA-MB-231 cells derived from MDA-MB-231 cells, from triple negative breast cancers (TNBCs), and evaluated whether RT-R-MDA-MB-231 cells increased the levels of HIF-1α and LOX. TNBC is a subtype of breast cancer characterized by a deficiency of expression of progesterone, estrogen, and epidermal growth factor receptor, and the most aggressive cancer among other breast cancer subtypes due to the lack of therapeutic target.

According to several reports, hypoxia is attributed to the HIF-dependent expression of numerous genes, including Oct-4, Notch, and ALDH1, which play roles in regulating CSC populations and stemness [40,41,42,43,44], and promoting tumor relapse after therapy [45]. In our previous study [24] and the present study, RT-R-MDA-MB-231 cells induced HIF-1α expression both in vitro [24] and in vivo, and CSCs markers such as Oct3/4, Notch-4, and ALDH1 in vitro [24], and Oct3/4 and Notch-4 in vivo, respectively. Therefore, we suggest that RT-R-MDA-MB-231 cells increase CSCs through induction of HIF-1α-dependent expression of CSC markers such as Oct3/4, Notch-4, and/or ALDH1. Interestingly, in contrast to our in vitro study, which showed that RT-R-MDA-MB-231 cells significantly increased the expression of CSC markers CD44, Notch4, and OCT3/4 including ALDH1 compared to MDA-MB-231 [24]. Tumor tissue in RT-R-MDA-MB-231-injected mice showed significant increases in the expression of CD44, Notch-4, and Oct3/4, but not ALDH1 in the present study. This result corresponded with the clinical study in patients with breast cancer, where CD44, Oct3/4, and Notch-4, but not ALDH1A1, were significantly expressed in the tumor tissues compared with the normal epithelial tissues of patients with breast cancer [46]. These results suggest that the phenotype of cancer cells can change under different microenvironments in vivo, as supported by Prasetyanti and Medema [47].

Moreover, RT-R-MDA-MB-231-injected mice showed significantly increased plasma LOX levels and a higher level of CD11b^+^ BMDC recruitment around the sites of crosslinked collagen in the lungs compared with mice injected with MDA-MB-231 cells, suggesting that RT-R-MDA-MB-231 cells stimulate premetastatic niche formation in vivo through activation of the HIF-1α-LOX axis. LOX is known as an enzyme that crosslinks extracellular matrix proteins such as collagen and promotes breast cancer metastasis, and the release of LOX is regulated by HIF-1α [38]. However, in this study, we failed to observe metastasis to the lungs in mice injected with RT-R-MDA-MB-231 or MDA-MB-231 cells, perhaps because the experimental conditions differed from those in our previous study [48]. In the present study, we used NOD-SCID (deficient for B and T cells but not NK cells) with the expectation that these more severely immunodeficient mice would show obvious metastases; however, we did not observe metastases even in RT-R-MDA-MB-231 cell-injected mice. The tumors in mice injected with RT-R-MDA-MB-231 cells showed recurrence and faster growth than mice injected with MDA-MB-231 cells, but these disappeared during the early phase. We assume that NK cells might be important for mitigating cancer cell activity, and CSCs have the ability to promote disease recurrence. Because the premetastatic niche was formed in the lungs of both groups of mice, albeit to a greater extent in the mice injected with RT-R-MDA-MB-231 cells, we plan to further study the effects of NK cells on CSC-mediated metastasis and recurrence.

## 4. Conclusions

**Figure 6:**
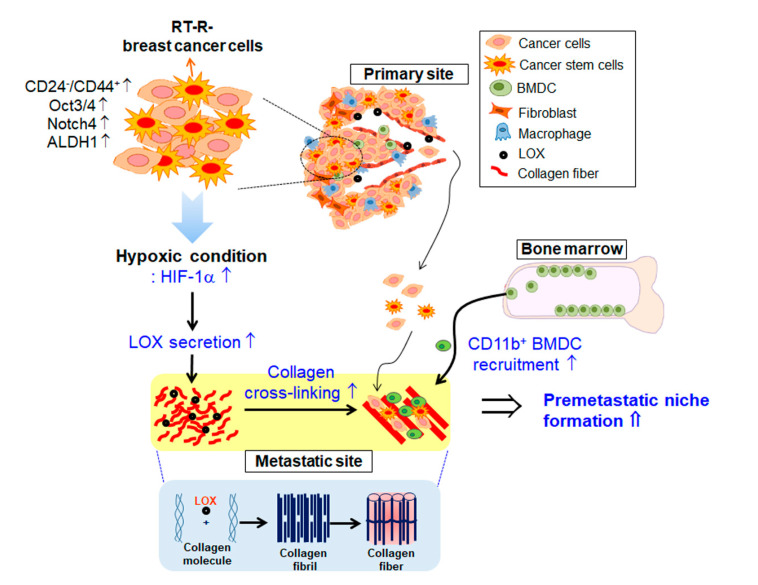
Schematic representation by which RT-R-breast cancer cells increase HIF-1α expression, leading to secretion of LOX, which contributes to tumor progression by enhancing premetastatic niche formation.

Taken together, this study suggests that RT-R-MDA-MB-231 cells derived from highly metastatic TNBCs increase HIF-1α expression, leading to secretion of LOX, which contributes to tumor progression by enhancing premetastatic niche formation (Figure 6).

## 5. Materials and Methods

### 5.1. Materials

RPMI 1640 medium, fetal bovine serum (FBS), and antibiotics (penicillin/streptomycin) were purchased from HyClone (South Logan, UT, USA). Anti-HIF-1α, anti-LOX, anti-Oct3/4, and anti-Notch-4 antibodies were purchased from Santa Cruz Biotechnology (Santa Cruz, CA, USA). Anti-CD11b and anti-ALDH1 antibodies were purchased from Abcam (Cambridge, UK). Enhanced chemiluminescence (ECL) western blotting detection reagent was obtained from Bio-Rad (Hercules, CA, USA). 3,3′-Diaminobenzidine tetrahydrochloride (DAB) immunostaining detection reagent was obtained from Thermo Fisher Scientific (Waltham, MA, USA). All other chemicals were purchased from Sigma-Aldrich (St. Louis, MO, USA).

### 5.2. Cell Culture and Establishment of RT-R-Breast Cancer Cells

The human breast cancer cell line MDA-MB-231 was obtained from the Korea Cell Line Bank (Seoul, Korea) and grown in RPMI 1640 supplemented with 10% FBS, 1% penicillin, and streptomycin. RT-R-MDA-MB-231 cells were generated by applying the cells with fractionated small doses (2 Gy) of X-ray irradiation until a final dose of 50 Gy was reached as described by Ko et al. [24]. The generation of RT-R-MDA-MB-231 cells was confirmed based on cell viability after exposure to radiation treatment [24]. RT-R-MDA-MB-231 cells were used through 5 passages.

### 5.3. Cell Proliferation Assay

Cells were seeded at 10^4^ cells per well in 24-well plates. Cells were incubated at 37 °C for 24, 48, and 72 h. After removal of media, a new medium containing 0.1 mg/mL MTT was added onto the cells and incubated for an additional 3 h. Then, the supernatants were aspirated, and the formazan crystals were dissolved with 200 µL of 4 N HCl-isopropanol in each well. The optical density of the colored product was measured at 570 nm, as suggested by the manufacturer, using an Infinite 200 microplate reader (TECAN, GmbH, Grödig, Austria).

### 5.4. Colony Formation Assay

Cells were seeded in 6-well plates at 10^3^ cells/well. Culture medium was changed with new media every 2–3 days. After 10 days, the medium was discarded, and each well was washed with phosphate buffered saline (PBS). The colonies were fixed in 100% methanol for 10 min at room temperature and then stained with 0.1% Giemsa staining solution for 30 min at room temperature and photographed.

### 5.5. Western Blot Analysis

Cells were harvested and lysed with a radioimmunoprecipitation assay (RIPA)buffer containing 50 mM Tris–HCl (pH 7.5), 150 mM NaCl, 1% NP-40, 0.1% SDS, 0.5% sodium deoxycholate, and protease inhibitors. The samples were centrifuged at 13000 rpm for 20 min at 4 °C, and the supernatants were collected for determination of the protein concentration using the Bradford method. Aliquots of 30–50 μg of protein were subjected to 8–10% sodium dodecyl sulfate–polyacrylamide gel electrophoresis (SDS-PAGE) gel for 2 h at 100 V. For the detection of secreted LOX in medium, proteins in conditioned media were concentrated 20-fold with Pierce concentrator 7 mL/9K, MWCO devices (Thermo Fisher Scientific). The separated proteins in the SDS-polyacrylamide gel were transferred onto Hybond-P^+^ polyvinylidene difluoride membranes (GE Healthcare, Little Chalfont, UK). The membranes were blocked with 5% nonfat milk in Tris-buffered saline containing 0.05% Tween-20 for 1 h at room temperature and then incubated with the primary antibodies: anti-HIF-1α (ab2185; 1:1000, Abcam) and anti-LOX (sc-32409; 1:1000, Santa Cruz Biotechnology, Dallas, TX, USA) antibodies. The bound antibodies were detected with horseradish peroxidase-conjugated secondary antibodies and an enhanced chemiluminescence (ECL) western blotting detection reagent.

### 5.6. Animal Experiments

Female NOD-SCID mice (Animal Resources Center; ARC, Australia) were divided into 2 groups (10 mice/group), and injected subcutaneously into the mammary fat pad with MDA-MB-231 cells and RT-R-MDA-MB-231 cells (5 × 10^6^ cells/100 μL of serum-free medium), respectively. The experimental protocol was approved by the Institutional Animal Care and Use Committee at Gyeongsang National University (approval number: GLA-120208-M004). Body weights and tumor volumes were measured every 3 days starting 7 days after injection. At the end of 90 days, the mice were sacrificed. The plasma LOX levels in mice injected with RT-R-MDA-MB-231 or MDA-MB-231 cells were determined by western blot analysis. Lung and tumor tissues were fixed in 10% formalin at room temperature, followed by paraffin infiltration and embedding. Sections of 5 μm were mounted onto gelatin-coated slides, and immunohistochemical analysis was performed using anti-CD44 (ab51037; 1:100, Abcam), anti-CD24 (sc-11406; 1:100, Santa Cruz Biotechnology), anti-Oct3/4 (sc-9081; 1:100, Santa Cruz Biotechnology), anti-Notch-4 (sc-5594; 1:100, Santa Cruz Biotechnology), anti-ALDH1 (ab52492; 1:100, Abcam), and anti-HIF-1α (ab2185; 1:100, Abcam) antibodies. After antibody staining, the sections were counterstained with hematoxylin and examined under a light microscope. Picrosirius Red (Sigma–Aldrich) was used for staining fibrillar collagen. After staining with the CD11b antibody (ab8878; 1:100, Abcam) and Picrosirius Red, sections were counterstained with hematoxylin. Representative areas of Picrosirius Red-stained lung sections were imaged under a polarized microscope (BX-51P, Olympus, Tokyo, Japan). The expression of the parameters under examination (CD24, CD44, Notch-4, Oct3/4 ALDH1, and HIF1α) was evaluated by counting the cells stained with each antibody and presenting the percentage of stained cells with each antibody in the tumor region.

### 5.7. Statistical Evaluations

Scanning densitometry was performed using Image Master^®^ VDS (Pharmacia Biotech Inc., San Francisco, CA, USA). The treatment groups were compared using one-way analysis of variance and Tukey’s multiple comparisons test. The data are presented as the mean ± standard error (SEM).

## Abbreviations

ALDH1	aldehyde dehydrogenase 1
BCSC	breast cancer stem cell
BMDCs	bone marrow-derived dendritic cells
CSC	cancer stem cell
ECL	enhanced chemiluminescence
FBS	fetal bovine serum
HIF	hypoxia inducible factor
LOX	lysyl oxidase
MTT	3-(4,5-dimethylthiazol-2-yl)-2,5-biphenyl tetrazolium bromide
PBS	phosphate-buffered saline
NOD-SCID	non obese diabeticsevere combined immunodeficiency
SDS-PAGE	sodium dodecyl sulfate-polyacrylamide gel electrophoresis
SEM	standard error
RIPA	Radioimmunoprecipitation assay
RT-R	radiotherapy-resistant
TNBC	triple negative breast cancer
